# Use of Bayes factors to evaluate the effects of host genetics, litter and cage on the rabbit cecal microbiota

**DOI:** 10.1186/s12711-022-00738-2

**Published:** 2022-06-27

**Authors:** María Velasco-Galilea, Miriam Piles, Yuliaxis Ramayo-Caldas, Luis Varona, Juan Pablo Sánchez

**Affiliations:** 1 Institute of Agrifood Research and Technology (IRTA)-Animal Breeding and Genetics, Caldes de Montbui, Barcelona, Spain; 2 Centre for Research in Agricultural Genomics (CRAG), CSIC-IRTA-UAB-UB, Bellaterra, Barcelona Spain; 3 Veterinary Faculty, University of Zaragoza, Saragossa, Spain

## Abstract

**Background:**

The rabbit cecum hosts and interacts with a complex microbial ecosystem that contributes to the variation of traits of economic interest. Although the influence of host genetics on microbial diversity and specific microbial taxa has been studied in several species (e.g., humans, pigs, or cattle), it has not been investigated in rabbits. Using a Bayes factor approach, the aim of this study was to dissect the effects of host genetics, litter and cage on 984 microbial traits that are representative of the rabbit microbiota.

**Results:**

Analysis of 16S rDNA sequences of cecal microbiota from 425 rabbits resulted in the relative abundances of 29 genera, 951 operational taxonomic units (OTU), and four microbial alpha-diversity indices. Each of these microbial traits was adjusted with mixed linear and zero-inflated Poisson (ZIP) models, which all included additive genetic, litter and cage effects, and body weight at weaning and batch as systematic factors. The marginal posterior distributions of the model parameters were estimated using MCMC Bayesian procedures. The deviance information criterion (DIC) was used for model comparison regarding the statistical distribution of the data (normal or ZIP), and the Bayes factor was computed as a measure of the strength of evidence in favor of the host genetics, litter, and cage effects on microbial traits. According to DIC, all microbial traits were better adjusted with the linear model except for the OTU present in less than 10% of the animals, and for 25 of the 43 OTU with a frequency between 10 and 25%. On a global scale, the Bayes factor revealed substantial evidence in favor of the genetic control of the number of observed OTU and Shannon indices. At the taxon-specific level, significant proportions of the OTU and relative abundances of genera were influenced by additive genetic, litter, and cage effects. Several members of the genera *Bacteroides* and *Parabacteroides* were strongly influenced by the host genetics and nursing environment, whereas the family *S24-7* and the genus *Ruminococcus* were strongly influenced by cage effects.

**Conclusions:**

This study demonstrates that host genetics shapes the overall rabbit cecal microbial diversity and that a significant proportion of the taxa is influenced either by host genetics or environmental factors, such as litter and/or cage.

**Supplementary Information:**

The online version contains supplementary material available at 10.1186/s12711-022-00738-2.

## Background

The bacterial communities that inhabit the rabbit gastrointestinal tracts play a key role in its metabolism, nutrition, and state of the immune system [1]. In the particular case of this herbivorous mammal, the richest and most diverse microbial community lies in its cecum [2]. Rabbit cecal microbial composition and diversity evolve from a simple and unstable community at birth into a complex and more stable one in the adult individuals [3]. Although this stability is reached at adulthood, previous studies have revealed that external factors, such as feed composition [4, 5], level of feeding [6, 7], or the administration of antibiotics [6–8] have a role in shaping the gut microbial composition and diversity. In addition to these environmental factors, host genetics could also play an important role. Several studies in humans [9, 10], cattle [11–15], pigs [16–19], and mice [20] have investigated the role of the host genetics on gut microbiota and have reported moderate heritabilities for certain microbial taxa and diversity indices. Thus, there is growing interest in investigating the interplay between the host genetics and the gut microbiota and its impact on many complex traits, such as human diseases, feed efficiency, or methane emissions in cattle.

In rabbit breeding, production traits such as feed efficiency and growth are key elements of economic profit [21]. Studies that attempt to unravel the existence of a potential link between these traits and the host genetics and microbiota are of great relevance for the rabbit industry to define effective genetic selection and production strategies that contribute to sustainable production and animal well-being. In this respect, previous studies have reported associations between the gut microbiota and growth [22] or feed efficiency in rabbits [23]. In addition, a substantial percentage of the phenotypic variance of growth, feed intake and feed efficiency in growing rabbits has been attributed to variation in their cecal microbiota [24]. However, to initiate selective breeding for the presence of microbial taxa that are positively associated with relevant traits, the genetic background of the rabbit cecal microbiota needs to be dissected. Velasco-Galilea et al. [24] has already provided some indirect evidence of host genetics control of the rabbit cecal microbiota since the predictive value of the microbial information for feed efficiency and other performance traits can be partially explained by the host additive genetic effects. Nonetheless, it is necessary to explicitly assess whether an overall host genetics control of microbiota exists or, on the contrary, whether only certain taxa or operational taxonomic units (OTU) are influenced by genetic effects. Moreover, to design effective breeding programs based on microbial information, it is necessary to know whether heritable taxa are associated with relevant production traits.

Many OTU are only present in a small percentage of the microbiota samples, which implies overdispersion due to an excessive number of zero counts that is not appropriately adjusted with a linear model. Thus, a zero-inflated Poisson (ZIP) model might be more suitable to estimate the heritability for these traits [25]. In a ZIP model, a given OTU is either not observed (zero counts) with probability $p$ or observed with a number of counts coming from a Poisson distribution with parameter $\lambda$ (the mean number of observations) with probability $1-p$.

Therefore, the objective of our study was to dissect the effects of the host genetics, litter and cage on a set of 984 microbial traits that were defined so that they represent the rabbit cecal microbiota at different levels of complexity (i.e., relative abundances of 29 genera, 951 normalized OTU, and four microbial alpha-diversity indices) in a meat rabbit population raised under standard commercial conditions. These traits were analyzed using Bayesian linear and ZIP mixed models, and the statistical relevance of the ratios of the different variance components to the phenotypic variance estimates was evaluated through Bayes factors (BF).

## Methods

### Animals

In total, 425 meat rabbits from the Caldes line [26] were included in this study that was conducted at the Institute of Agrifood Research and Technology (IRTA). Of these 425 rabbits, 336 were produced in four batches and housed in collective cages, each containing eight kits, in a semi-open-air facility during the first semester of 2014, and 89 were produced in a single batch and housed in collective cages, each containing six kits, in another facility under better controlled environmental conditions during the spring of 2016. After weaning (32 days of age), all the animals were kept under the same management conditions and were fed with a standard pelleted diet supplemented with antibiotics, except 23 rabbits from the second facility that received a diet free of antibiotics. The fattening period lasted 5 and 4 weeks for the animals raised in the first and the second facility, respectively, and during the last fattening week, all the animals received an antibiotic-free diet. Water was supplied *ad libitum* and food was provided once per day in a feeder with three places. After weaning, kits were classified into two groups according to their size (“big” if their body weight was greater than 700 g or “small” otherwise) and randomly assigned to *ad libitum* (AL) feeding regime or a regime restricted (R) to 75% of the AL feed intake. The amount of feed supplied to the animals under R for each week and each batch was computed as 0.75 times the average feed intake of kits on AL from the same batch during the previous week, plus 10% to account for a feed intake increase as the animal grows. To prevent a possible association between cage and maternal effects, a maximum of two kits belonging to the same litter were assigned to the same cage. The dataset used for this study included information on 425 individuals born from 196 litters and housed in 189 cages. The pedigree included information on 9760 individuals, tracing back 20 generations of ancestors up to animals born in 2005.

### Sample collection, DNA extraction and sequencing

At slaughter, cecal samples from each animal were collected in a sterile tube, first kept at 4 °C in the laboratory, and then stored at − 80 °C. Extraction and amplification of DNA, Illumina library preparation and sequencing were performed as described in [6]. To facilitate efficient lysis, 250 mg of each sample were mechanically lysed in a FastPrep-24TM Homogenizer (MP Biomedicals, LLC, Santa Ana, CA, USA) at a speed of 6 m/s for 60 s. The kit ZR Soil Microbe DNA MiniPrepTM (ZymoResearch, Freiburg, Germany) was used to extract whole genomic DNA. The integrity and purity of the DNA were measured with a Nanodrop ND-1000 spectrophotometer (NanoDrop products; Wilmington, DE, USA) following the protocol of Desjardins and Conklin [27]. The F515Y/R926 pair of primers (5′-GTGYCAGCMGCCGCGGTAA-3′, 5′-CCGYCAATTYMTTTRAGTTT-3′) [28] was used to amplify a fragment of the 16S rRNA gene that included the V4-V5 hypervariable regions. An initial polymerase chain reaction (PCR) was conducted for each sample with 12.5 µL 2× KAPA HiFi HotStart Ready Mix, 5 µL forward primer, 5 µL reverse primer and 2.5 µL template DNA (5 ng/ µL) under the following conditions: initial denaturation for 3 min at 95 °C, 25 cycles of 30 s at 95 °C, 30 s at 55 °C and 30 s at 72 °C; and final extension for 2 min at 72 °C. Then, sequencing adaptors and eight nucleotide dual-indexed barcodes of the multiplex Nextera^®^ XT kit (Illumina, Inc., San Diego CA, USA) were added in a second PCR reaction with 25 µL 2× KAPA HiFi HotStart Ready Mix, 5 µL index i7, 5 µL index i5, 10 µL PCR Grade water and 5 µL concentrated amplicons of the initial PCR. Conditions for this second PCR were: an initial denaturation for 3 min at 95 °C, 8 cycles of 30 s at 95 °C, 30 s at 55 °C and 30 s at 72 °C; and a final extension for 5 min at 72 °C. The libraries obtained were cleaned up with AMPure XP beads, validated by running 1 µL of a 1:50 dilution on a Bioanalyzer DNA 1000 chip (Agilent Technologies, Inc., Santa Clara, CA, USA) to verify their size, and quantified by fluorometry with the PicoGreen dsDNA quantification kit (Invitrogen, Life Technologies, Carlsbad, CA, USA). After size verification, libraries were pooled at equimolar concentrations and paired-end sequenced in five parallel plates on an Illumina MiSeq 2× 250 platform at the Genomics and Bioinformatics Service of the Autonomous University of Barcelona.

### Bioinformatics processing of microbial traits

The pipeline of the QIIME software (version 1.9.0) [29] that was used for sequence processing is fully described in [6]. Briefly, paired-end reads were assembled into contigs using the python script *multiple_join_paired_ends.py* with default parameters. Then, the contigs with a quality score lower than Q19 were discarded, and the remaining ones were assigned to samples using the python script *split_libraries.py* with default parameters. The UCHIME algorithm [30] was used to detect and remove the chimeric sequences generated during PCR. The filtered contigs were clustered into OTU with a 97% similarity threshold using the script *pick_open_reference_otus.py* with default parameters [31] and the Greengenes reference database (version gg_13_5_otus) [32]. The obtained OTU table was normalized with the cumulative sum scaling (CSS) method [33]. Finally, the UCLUST consensus taxonomy assigner was used to conduct the taxonomic assignment of representative sequences of each OTU (the most abundant sequence of each cluster) by mapping the sequences against the Greengenes reference database gg_13_5_otus. The raw sequence data were deposited in the sequence read archive of NCBI under the BioProject accession number PRJNA524130. The metadata, OTU table, and corresponding taxonomic assignments are in Additional file 1: Table S1, Additional file 2: Table S2 and Additional file 3: Table S3, respectively (see Additional file 1: Table S1, Additional file 2: Table S2 and Additional file 3: Table S3). These data are also available in Qiita (https://qiita.ucsd.edu/) under study ID 14485. After the bioinformatic processing, 984 representative traits of the rabbit intestinal microbiota were defined and analyzed in the present study. These microbial traits can be categorized into three groups: the relative abundances of 29 genera, 951 CSS-normalized OTU, and four microbial alpha-diversity indices computed at 10,000 contigs (total number of OTU observed, Chao1, Shannon and Simpson's inverse). Relative abundances of genera and microbial alpha-diversity indices were standardized by subtracting their mean and dividing by their standard deviation. Finally, these standardized microbial traits and CSS-normalized OTU were multiplied by 100 and subsequently rounded to the nearest integer. This transformation was necessary to generate integers that can be treated as counts, thus enabling the adjustment of a ZIP model.

### Statistical models

#### Zero-inflated Poisson (ZIP) mixed model

Let $\mathbf{y}=\left({\mathrm{y}}_{1}, {\mathrm{y}}_{2},\dots , {\mathrm{y}}_{\mathrm{n}}\right)\mathrm{^{\prime}}$ be the vector of the records of a specific microbial trait on $\mathrm{n}$ individuals. Zero-inflation occurs with probability p, and data for animal $\mathrm{i}$ follow a Poisson distribution with parameter ${\uplambda }_{\mathrm{i}}$ occur with probability $\left(1-\mathrm{p}\right)$. Thus, the probability of observing a zero count is $\mathrm{p}\left({\mathrm{y}}_{\mathrm{i}}=0\right)=\mathrm{p}+\left(1-\mathrm{p}\right){\mathrm{e}}^{{-\uplambda }_{\mathrm{i}}}$, and the probability of observing a count of $\mathrm{k}$ ($\mathrm{k}$ = 1, 2, …, ∞) is $\mathrm{p}\left({\mathrm{y}}_{\mathrm{i}}=\mathrm{k}\right)=\frac{\left(1-\mathrm{p}\right){\mathrm{e}}^{{-\uplambda }_{\mathrm{i}}}{\uplambda }_{\mathrm{i}}^{\mathrm{k}}}{\mathrm{k}!}$. Therefore, $\mathrm{p}$ is a population parameter while ${\uplambda }_{\mathrm{i}}$ is an animal-specific parameter. Conditioning on both $\mathrm{p}$ and ${\varvec{\uplambda}}$, which is the vector of the ${\uplambda }_{\mathrm{i}}$ of all the individuals, the likelihood function can be expressed as follows:

1 $$\mathrm{p}\left(\mathbf{y}|{\varvec{\uplambda}},\mathrm{p}\right)=\prod_{{\mathrm{y}}_{\mathrm{i}}=0}\left[\mathrm{p}+\left(1-\mathrm{p}\right){\mathrm{e}}^{-{\uplambda }_{\mathrm{i}}}\right]\prod_{{\mathrm{y}}_{\mathrm{i}}>0}\left[\frac{\left(1-\mathrm{p}\right){\mathrm{e}}^{-{\uplambda }_{\mathrm{i}}}{{\uplambda }_{\mathrm{i}}}^{{\mathrm{y}}_{\mathrm{i}}}}{{\mathrm{y}}_{\mathrm{i}}!}\right].$$

Considering these two re-parameterizations:

2 $${\uplambda }_{\mathrm{i}}^{*}=\mathrm{log}\left({\uplambda }_{\mathrm{i}}\right),$$

3 $${\mathrm{p}}^{*}=\mathrm{log}\left(\frac{\mathrm{p}}{1-\mathrm{p}}\right),$$

the previous conditional likelihood can be expressed as:

4 $$\mathrm{p}\left(\mathbf{y}|{{\varvec{\uplambda}}}^{\mathbf{*}},{\mathrm{p}}^{*}\right)=\prod_{{\mathrm{y}}_{\mathrm{i}}=0}\left[\left(\frac{1}{\left(1+ {\mathrm{e}}^{{\mathrm{p}}^{*}}\right)}\right)\left[{\mathrm{e}}^{{\mathrm{p}}^{*}}+{\mathrm{e}}^{-\mathrm{exp}\left({\uplambda }_{\mathrm{i}}^{*}\right)}\right]\right]\prod_{{\mathrm{y}}_{\mathrm{i}}>0}\left[\left(\frac{1}{\left(1+ {\mathrm{e}}^{{\mathrm{p}}^{*}}\right)}\right)\frac{{\mathrm{e}}^{-\mathrm{exp}\left({\uplambda }_{\mathrm{i}}^{*}\right)+{\uplambda }_{\mathrm{i}}^{*}{\mathrm{y}}_{\mathrm{i}}}}{{\mathrm{y}}_{\mathrm{i}}!}\right],$$

since $${\uplambda }_{\mathrm{i}}=\mathrm{exp}\left({\uplambda }_{\mathrm{i}}^{*}\right)$$ and $$\mathrm{p}=\frac{\mathrm{exp}\left({\mathrm{p}}^{*}\right)}{1+\mathrm{exp}\left({\mathrm{p}}^{*}\right)}$$.

At a subsequent hierarchical level, different factors can be included as a linear model to explain the vector ${{\varvec{\uplambda}}}^{\mathbf{*}}$, thus, the assumed distribution for ${{\varvec{\uplambda}}}^{\mathbf{*}}$ is the following normal density:

5 $$\mathrm{p}\left({{\varvec{\uplambda}}}^{\mathbf{*}}|\mathbf{V},{\varvec{\upbeta}}\right)\sim \mathrm{MVN}\left(\mathbf{X}{\varvec{\upbeta}},\mathbf{V}\right),$$

where ${\varvec{\upbeta}}$ is a vector of systematic factors including the effects of the different categories of the combination between breeding farm, diet, and feeding regime (6 levels), of batch (5 levels) and of body weight at weaning (2 levels). $\mathbf{X}$ is a design matrix that relates the observations to the systematic effects, and $\mathbf{V}$ is the covariance matrix between the elements of ${{\varvec{\uplambda}}}^{\mathbf{*}}$. The structure of $\mathbf{V}$ is not diagonal, and is defined as follows:

6 $$\begin{aligned}\mathbf{V} & = {\upsigma }_{\mathrm{P}}^{2}[{\mathbf{Z}}_{\mathbf{A}}\mathbf{A}{\mathbf{Z}}_{\mathbf{A}}^{\mathrm{^{\prime}}}{\mathrm{h}}^{2}+{\mathbf{Z}}_{\mathbf{L}}{\mathbf{Z}}_{\mathbf{L}}^{\mathrm{^{\prime}}}{\mathrm{l}}^{2}+{\mathbf{Z}}_{\mathbf{C}}{\mathbf{Z}}_{\mathbf{C}}^{\mathrm{^{\prime}}}{\mathrm{c}}^{2} \\ & \quad +\mathbf{I}\left(1-{\mathrm{h}}^{2}-{\mathrm{l}}^{2}-{\mathrm{c}}^{2}\right)],\end{aligned}$$

where ${\upsigma }_{\mathrm{P}}^{2}$ is the phenotypic variance and the scalars ${\mathrm{h}}^{2}$, ${\mathrm{l}}^{2}$ and ${\mathrm{c}}^{2}$ represent the ratios of additive genetic, litter and cage variances to ${\upsigma }_{\mathrm{P}}^{2}$. The assumed joint prior distribution of these ratios was uniform, with the constraint that their sum must be less than 1:

7 $$\mathrm{p}\left({\mathrm{h}}^{2}, {\mathrm{l}}^{2},{\mathrm{c}}^{2}\right)=6, \;\; \mathrm{ if }\;\;{\mathrm{ h}}^{2}+{\mathrm{l}}^{2}+{\mathrm{c}}^{2}\in \left[0, 1\right],\;\;\mathrm{ or }\;\;0\;\;\mathrm{ otherwise}.$$

To guarantee a density of 1, the joint prior distribution under such a constraint was set to 6. Note that the constraint implies that the density of the resulting distribution will be just 1/6 of the 3-variate uniform distribution without constraint. As explained later, the marginal prior distributions are needed to define the BF for the ratios of variance. Assuming prior independence, the marginal priors of ${\mathrm{h}}^{2}$, ${\mathrm{l}}^{2}$ and ${\mathrm{c}}^{2}$ from the constrained joint distribution are the following Beta distributions:

8 $$\mathrm{p}\left({\mathrm{h}}^{2}\right)=\mathrm{Beta}\left(\mathrm{1,3}\right)=3{\left({\mathrm{h}}^{2}\right)}^{2}-6{\mathrm{h}}^{2}+3,$$

9 $$\mathrm{p}\left({\mathrm{l}}^{2}\right)=\mathrm{Beta}\left(\mathrm{1,3}\right)=3{\left({\mathrm{l}}^{2}\right)}^{2}-6{\mathrm{l}}^{2}+3,$$

10 $$\mathrm{p}\left({\mathrm{c}}^{2}\right)=\mathrm{Beta}\left(\mathrm{1,3}\right)=3{\left({\mathrm{c}}^{2}\right)}^{2}-6{\mathrm{c}}^{2}+3.$$

A uniform distribution along the positive real numbers was also assumed for ${\upsigma }_{\mathrm{P}}^{2}$. ${\mathbf{Z}}_{\mathbf{A}}$, ${\mathbf{Z}}_{\mathbf{L}}$ and ${\mathbf{Z}}_{\mathbf{C}}$ are design matrices relating the observations with animals in the pedigree (9760 levels), litters (196 levels) and cages (189 levels), respectively, and $\mathbf{A}$ is the numerator relationship matrix [34]. Uniform priors were assumed for the elements of ${\varvec{\upbeta}}$ and ${\mathrm{p}}^{*}$, bounded between − 5 and + 5 for the latter.

The posterior density can be written as:

$$\mathrm{p}({{\varvec{\uplambda}}}^{\mathbf{*}},{\mathrm{p}}^{*},\mathbf{V},{\varvec{\upbeta}}|\mathbf{y}) \propto \mathrm{p}(\mathbf{y}|{{\varvec{\uplambda}}}^{\mathbf{*}},{\mathrm{p}}^{*})\mathrm{p}\left({{\varvec{\uplambda}}}^{\mathbf{*}}|\mathbf{V},{\varvec{\upbeta}}\right)\mathrm{p}\left({\mathrm{p}}^{*}\right)\mathrm{p}\left(\mathbf{V}\right)\mathrm{p}\left({\varvec{\upbeta}}\right)$$

11 $$\begin{aligned} & \mathrm{p}\left({{\varvec{\uplambda}}}^{\mathbf{*}},{\mathrm{p}}^{*},\mathbf{V},{\varvec{\upbeta}}|\mathbf{y}\right) \\ & \quad \propto \prod_{{\mathrm{y}}_{\mathrm{i}}=0}\left[\left(\frac{1}{\left(1+ {\mathrm{e}}^{{\mathrm{p}}^{*}}\right)}\right)\left[{\mathrm{e}}^{{\mathrm{p}}^{*}}+{\mathrm{e}}^{-\mathrm{exp}\left({\uplambda }_{\mathrm{i}}^{*}\right)}\right]\right] \\ & \qquad \times \prod_{{\mathrm{y}}_{\mathrm{i}}>0}\left[\left(\frac{1}{\left(1+ {\mathrm{e}}^{{\mathrm{p}}^{*}}\right)}\right)\frac{{\mathrm{e}}^{-\mathrm{exp}\left({\uplambda }_{\mathrm{i}}^{*}\right)+{\uplambda }_{\mathrm{i}}^{*}{\mathrm{y}}_{\mathrm{i}}}}{{\mathrm{y}}_{\mathrm{i}}!}\right] \\ & \qquad \times {\left|\mathbf{V}\right|}^{\mathrm{n}/2}\mathrm{exp}\left\{-\frac{1}{2}\left({{\varvec{\uplambda}}}^{\mathbf{*}}-\mathbf{X}{\varvec{\upbeta}}\right)\mathrm{^{\prime}}{\left(\mathbf{V}\right)}^{-1}\left({{\varvec{\uplambda}}}^{\mathbf{*}}-\mathbf{X}{\varvec{\upbeta}}\right)\right\}\end{aligned}$$

This model specification is similar to that previously proposed for studying mastitis cases in dairy cows [35]. The differences introduced in our study refer to the specifications for ${{\varvec{\uplambda}}}^{\mathbf{*}}$. In our case, we assume a model in which a number of factors was absorbed into the residual, while these factors were explicitly fitted in the model of Rodrigues-Motta et al. [35] as part of the vector of the means. Apart from these differences in the prior assumption, the two models are equivalent. The parameterization used here allows the computation of the BF for the ratio of variances in a parametric space defined between 0 and 1, including both limits, although it is more computationally demanding than that of Rodrigues-Motta et al. [35]. The reasons are that, first, $\mathbf{V}$ must be updated and inverted repeatedly and second, Metropolis-Hasting steps are needed to update the conditional posterior distribution of the ratios. In contrast to the case where the effects are explicitly considered in the model [35], the BF can be computed to test whether the additive genetic, litter, and cage effects are null or not, since this model parameterization allows a null value of the ratio. The derivation of the conditional posterior distributions can be followed in the studies of Rodrigues-Motta et al. [35] and Varona et al. [36] on which our model is based on.

#### Linear mixed model (LMM)

This model can be considered as a simplification of the previous one since the generation process assumed for all the data was the same as that assumed for the logarithm of the vector of $\uplambda$ parameters of the individual Poisson distributions (${{\varvec{\uplambda}}}^{\mathbf{*}}$) corresponding to the records with non-zero counts for each trait (transformed CSS-normalized OTU counts, transformed relative abundances of genera, and transformed alpha-diversity indices). Thus, the distribution of the data given the model parameters can be written as:

12 $$\mathrm{p}\left(\mathbf{y}|\mathbf{V},{\varvec{\upbeta}}\right)\sim \mathrm{MVN}\left(\mathbf{X}{\varvec{\upbeta}},\mathbf{V}\right).$$

Notation and model specifications, including both the structure of $\mathbf{V}$ (Eq. (6)) and the *prior* definitions (Eq. (7)), were assumed to be the same as in the ZIP model, except for the parameters that are specific to the ZIP model. For implementation, we used the conditional posterior distributions of this LMM derived by Varona et al. [36] since we assumed the same *prior* distributions as theirs.

#### Criteria for comparison of models

Two criteria for model choice were applied for each of the 984 microbial traits analyzed in this study. First, we evaluated whether the trait was better adjusted with the LMM or the ZIP model with the preferred model being that with the lowest deviance information criterion (DIC) value [37]. The statistical relevance of the additive genetic, litter and cage effects was evaluated for each trait with the model that best fitted the trait (either LMM or ZIP) using the BF. Thus, for each trait, three BF were computed to assess the null hypotheses of whether the additive genetic (a), litter (l) or cage (c) effects have a null effect versus the alternative hypothesis that assumed that these factors have a non-null effect. These three hypotheses were independently tested by computing the BF of ${\mathrm{h}}^{2}=0$ against ${\mathrm{h}}^{2}\ne 0$ (${\mathrm{BF}}_{{\mathrm{h}}^{2}}$), ${\mathrm{l}}^{2}=0$ against ${\mathrm{l}}^{2}\ne 0$ (${\mathrm{BF}}_{{\mathrm{l}}^{2}}$), and ${\mathrm{c}}^{2}=0$ against ${\mathrm{c}}^{2}\ne 0$ (${\mathrm{BF}}_{{\mathrm{c}}^{2}}$).

13 $${\mathrm{BF}}_{{\mathrm{h}}^{2}}=\frac{3}{\mathrm{p}\left({\mathrm{h}}^{2}=0 |\mathbf{y}\right)};\;\;{\mathrm{BF}}_{{\mathrm{l}}^{2}}=\frac{3}{\mathrm{p}\left({\mathrm{l}}^{2}=0 |\mathbf{y}\right)};\;\;\mathrm{and } \;\;{\mathrm{BF}}_{{\mathrm{c}}^{2}}=\frac{3}{\mathrm{p}\left({\mathrm{c}}^{2}=0 |\mathbf{y}\right)}$$

The derivations of these definitions of the BF are reported in Varona et al. [36]. They show how the BF for nested models is the ratio between the marginal prior density evaluated at the value of interest, zero in our case, divided by the marginal posterior density at the value of interest (i.e., for the heritability: ${\mathrm{BF}}_{{\mathrm{h}}^{2}}=\frac{\mathrm{p}\left({\mathrm{h}}^{2}=0 \right)}{\mathrm{p}\left({\mathrm{h}}^{2}=0 |\mathbf{y}\right)}$). The marginal prior densities under our constrained joint prior distribution of the ratios are Beta(1,3) distributions, which are equal to 3 at the 0 value for the ratio of variance (Eq. (8)). The evaluation of the marginal posterior of the ratios at 0 implies that, since these marginal posteriors are only defined up to proportionality, the computation of this proportionality is constant: ${\int }_{{\mathrm{h}}^{2}=0}^{{\mathrm{h}}^{2}=1}\mathrm{p}\left({\mathrm{h}}^{2} |\mathbf{y}\right){\mathrm{dh}}^{2}$. This integral can be solved numerically at each iteration. The different BF can be computed as follows from the Markov chain Monte Carlo (MCMC) output:

14 $${\mathrm{BF}}_{{\mathrm{h}}^{2}}=\frac{3}{\sum_{\mathrm{j}=1}^{\mathrm{N}}\frac{{\mathrm{p}\left({\mathrm{h}}^{2}=0 |\mathbf{y}\right)}_{\mathrm{j}}}{\mathrm{N}}},$$

where $\mathrm{N}$ is the number of MCMC iterations and ${\mathrm{p}\left({\mathrm{h}}^{2}=0 |\mathbf{y}\right)}_{\mathrm{j}}$ is the evaluation of the marginal posterior density of ${\mathrm{h}}^{2}$ at zero at each iteration $\mathrm{j}$ of the sampling procedure, which is computed as stated above:

15 $$\frac{{\mathrm{p}\left({\mathrm{h}}^{2}=0 |\mathbf{y}\right)}_{\mathrm{j}}}{{\int }_{{\mathrm{h}}^{2}=0}^{{\mathrm{h}}^{2}=1}{\mathrm{p}\left({\mathrm{h}}^{2} |\mathbf{y}\right)}_{\mathrm{j}}{\mathrm{dh}}^{2}}.$$

All the operations were done on the logarithmic scale and after having saved the evaluations of the marginal posterior at zero along the MCMC chain to avoid numerical instabilities during their computation. In this way, it was possible to adjust the evaluations of the marginal posterior at zero for their maximum, thus reducing the needed numerical accuracy:

16 $$\sum_{\mathrm{j}=1}^{\mathrm{N}}\frac{{\mathrm{p}\left({\mathrm{h}}^{2}=0 |\mathbf{y}\right)}_{\mathrm{j}}}{\mathrm{N}}=\mathrm{exp}\left\{\mathrm{log}\left(\frac{\sum_{\mathrm{j}=1}^{\mathrm{N}}\mathrm{exp}\left\{\mathrm{log}\left({\mathrm{p}\left({\mathrm{h}}^{2}=0 |\mathbf{y}\right)}_{\mathrm{j}}\right)-\mathrm{m}\right\}}{\mathrm{N}}\right)+\mathrm{m}\right\},$$

where $\mathrm{m}$ is the maximum value of the vector composed of the $\mathrm{N}$ evaluations of $\mathrm{p}\left({\mathrm{h}}^{2}=0 |\mathbf{y}\right)$ on the logarithmic scale. See Sorensen and Gianola [38] for further details.

BF values were classified according to four levels of evidence [39]: BF < 3.2: the denominator model is supported; 3.2 $\le$ BF < 10: substantial evidence favoring the numerator model; 10 $\le$ BF < 100: strong evidence favoring the numerator model; and BF $\ge$ 100: decisive evidence favoring the numerator model. The reciprocal of the BF value (1/BF) was used to assess the evidence for the null hypothesis, which assumed that the additive genetic, litter or cage effects on the trait were null.

#### MCMC Bayesian implementation

MCMC Bayesian procedures were used to obtain samples from the marginal posterior distributions. This algorithm was implemented in a Fortran 90 software, which is available in our GitHub repository (https://github.com/juanpablo-sanchez/BF-ZIP). For both the LMM and ZIP models, chains of 10,000 samples were run discarding the first 1000 to allow the algorithm to reach convergence to the marginal posterior distributions. Convergence diagnostics of the Markov chains was performed by the Geweke test function with the coda R package [40]. The z-statistics of this test for the genetic, litter and cage variance ratios computed for the 984 microbial traits fitted with the LMM or ZIP models (according to DIC) are in Additional file 4: Fig. S1 and Additional file 5: Fig. S2, respectively. Although the computational demand of the parameterization on the variance ratios is high, it allows a good mixing. Thus, a reduced number of iterations was needed to properly reach convergence and characterize the marginal posterior distributions.

## Results

### Cecal microbial composition and diversity

After bioinformatic sequence processing, we identified 951 OTU that were present in at least 5% of the animals. Each sample had on average 677 OTU (range from 197 to 841). Table 1 shows the OTU frequencies across rabbit samples.

**Table 1:** OTU frequencies across rabbit cecal samples

Frequency (%)	Number of OTU
$\ge$ 5 to $\le$ 10	13
> 10 to $\le$ 25	43
> 25 to $\le$ 50	121
> 50 to $\le$ 75	277
> 75 to $\le$ 100	497

The taxonomic assignment of representative sequences of these OTU against the Greengenes reference database gg_13_5_otus (see Additional file 3: Table S3) revealed the presence of 29 known genera. Among these, four were present in 50 to 75% of the rabbit samples and 25 in a minimum of 75% of the animals. Table 2 shows a phenotypic summary of the relative abundances of the 29 genera together with the four microbial alpha-diversity indices computed.

**Table 2:** Phenotypic summary of genera and alpha diversity indices

Trait	Mean	SD
Genus *Methanobrevibacter*, %	0.19	0.23
Genus *Adlercreutzia*, %	0.95	0.43
Genus *Bacteroides*, %	1.65	0.76
Genus *Parabacteroides*, %	0.21	0.18
Genus *Rikenella*, %	0.35	0.24
Genus *Butyricimonas*, %	0.20	0.19
Genus *Odoribacter*, %	0.27	0.22
Genus *Clostridium*, %	1.05	0.26
Genus *Dehalobacterium*, %	0.08	0.09
Genus *Anaerofustis*, %	0.11	0.07
Genus *Anaerostipes*, %	0.16	0.08
Genus *Blautia*, %	2.94	0.65
Genus *Butyrivibrio*, %	0.11	0.07
Genus *Coprococcus*, %	2.02	0.42
Genus *Dorea*, %	0.47	0.12
Genus *Epulopiscium*, %	0.11	0.11
Genus *Ruminococcus*, %	0.16	0.07
Genus *rc4-4*, %	0.15	0.07
Genus *Faecalibacterium*, %	0.20	0.05
Genus *Oscillospira*, %	2.26	0.58
Genus *Phascolarctobacterium*, %	0.21	0.24
Genus *Coprobacillus*, %	0.19	0.24
Genus *p-75-a5*, %	0.10	0.07
Genus *Oxalobacter*, %	0.11	0.06
Genus *Desulfovibrio*, %	0.46	0.31
Genus *Campylobacter*, %	0.07	0.08
Genus *Ruminococcus*, %	4.32	0.85
Genus *Anaeroplasma*, %	0.20	0.17
Genus *Akkermansia*, %	1.47	0.50
Number of OTU observed	551.05	91.94
Shannon	5.07	0.30
Simpson	0.98	0.01
Simpson’s inverse	71.01	20.20

SD: standard deviation

### Adjustment of microbial traits with the LMM versus ZIP model

According to DIC, the adjustment of all relative abundances of genera and microbial alpha-diversity indices was better with the LMM (lower DIC values) than with the ZIP model (see Additional file 6: Table S4). Among the 951 CSS-normalized OTU also analyzed in our study, those with a frequency higher than 25% were better adjusted with the LMM and those with a frequency lower than 10% were better adjusted with the ZIP model. Among the 43 OTU with a frequency between 10 and 25%, 18 and 25 OTU were better adjusted with the LMM and the ZIP model, respectively (see Additional file 6: Table S4).

### Effects of the host genetics, litter and cage on the rabbit cecal microbiota

**Fig. 1:**
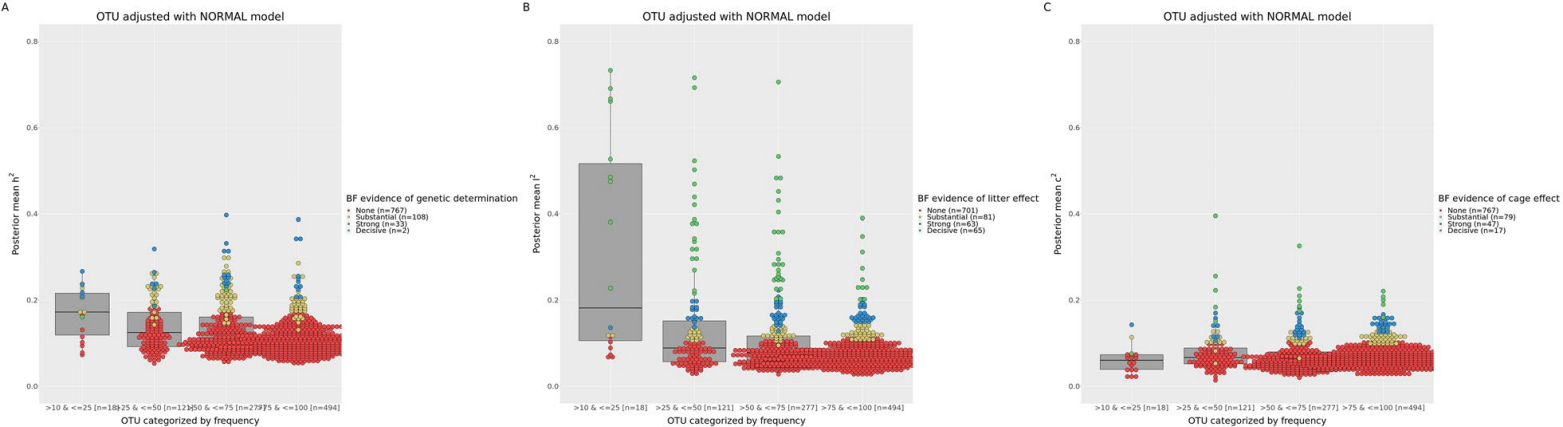
Marginal posterior of heritability (**a**), litter (**b**) and cage (**c**) variance ratios for OTU adjusted with the normal linear mixed model. OTU are categorized by their frequency among the rabbit samples. Each OTU is represented by a dot colored in red, yellow, blue or green based on Bayes factor for no, substantial, strong or decisive evidence, respectively, of additive genetic (**a**), litter (**b**) or cage (**c**) effects

**Fig. 2:**
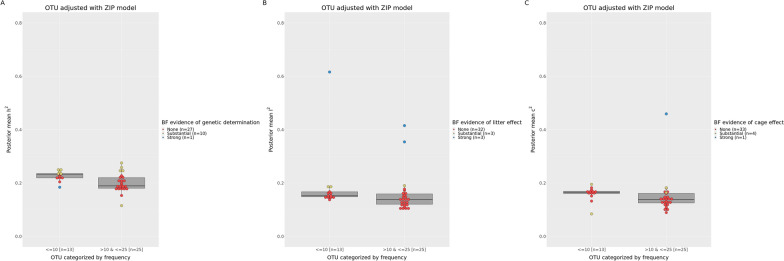
Marginal posterior of heritability (**a**), litter (**b**) and cage (**c**) variance ratios for OTU adjusted with the zero-inflated Poisson model. OTU are categorized by their frequency among the rabbit samples. Each OTU is represented by a dot colored in red, yellow, blue or green based on Bayes factor for no, substantial, strong or decisive evidence, respectively, of additive genetic (**a**), litter (**b**) or cage (**c**) effects

**Fig. 3:**
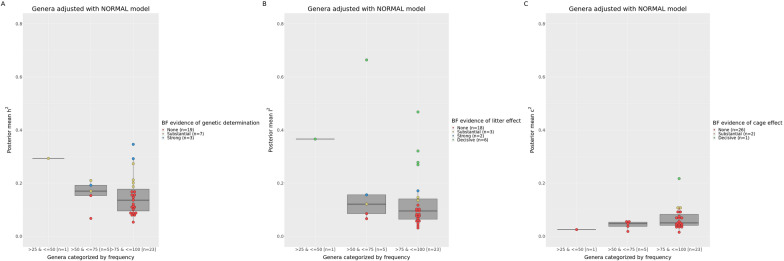
Marginal posterior of heritability (**a**), litter (**b**) and cage (**c**) variance ratios for relative abundances of genera adjusted with the normal linear mixed model. Genera are categorized by their frequency among the rabbit samples. Each genus is represented by a dot colored in red, yellow, blue or green based on Bayes factor for no, substantial, strong or decisive evidence, respectively, of additive genetic (**a**), litter (**b**) or cage (**c**) effects

**Fig. 4:**
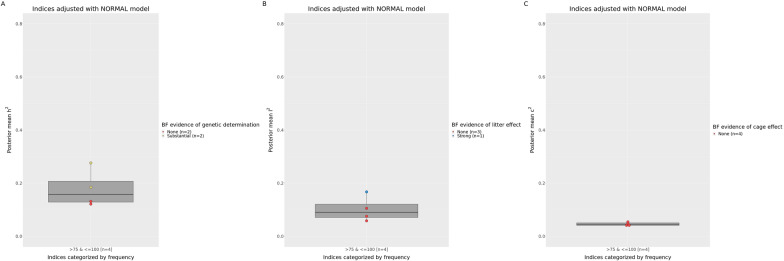
Marginal posterior of heritability (**a**), litter (**b**) and cage (**c**) variance ratios for microbial alpha-diversity indices adjusted with the normal linear mixed model. Each index is represented by a dot colored in red, yellow, blue or green based on Bayes factor for no, substantial, strong or decisive evidence, respectively, of additive genetic (**a**), litter (**b**) or cage (**c**) effects

Box and whisker plots of the estimated marginal posterior means of the heritability, and litter and cage variance ratios for the OTU that, according to DIC, are better adjusted with the LMM, and for those for which the ZIP model is preferable are shown in Figs. 1 and 2, respectively. The same plots corresponding to relative abundances of genera and microbial alpha-diversity indices are shown in Figs. 3, and 4, respectively. In all of the plots, microbial traits are categorized by their frequency among samples and by the BF that represent the levels of evidence in favor of the model that included additive genetic (a), litter (b), or cage (c) effects. Overall, these figures show that the BF did not provide evidence of genetic, litter, or cage effects for ~ 80% of the microbial traits analyzed. However, for the traits that were declared to be affected by the host genetics, litter or cage (see Additional file 7: Tables S5, S6 and S7, respectively), the magnitude of the estimates of variance ratios was moderate to high with minimum values of 0.15 to 0.20.

The results summarized in Figs. 1, 2, 3 and 4 are presented in the following paragraphs of the “Results” section. Bayes factors, marginal posterior means and standard deviations of variance ratios for the OTU that, based on the BF, were declared to be influenced by host genetics, litter, or cage effects (together with the associated probability of these estimates being greater than 0.10) are included in Additional file 8: Table S8 (heritable OTU), Additional file 9: Table S9 (OTU influenced by the nursing environment) and Additional file 10: Table S10 (OTU influenced by cage effects).

### Microbial traits under genetic control

Table 3 summarizes the marginal posterior means of the heritability estimates for the OTU, categorized by frequency, which were better adjusted with the normal LMM according to DIC and for which the BF provided evidence in favor of host genetics control. Similarly, Table 4 summarizes the marginal posterior means of the heritability estimates for OTU, categorized by frequency, which were better adjusted with the ZIP model according to DIC and for which the BF provided evidence in favor of host genetics control.

**Table 3:** Mean (standard deviation) of Bayes factors and heritability estimates for OTU under genetic control adjusted with the normal LMM

Frequency (%)	Substantial evidence of genetic influence (3.2 $\le$ ${\mathrm{BF}}_{{\mathrm{h}}^{2}}$ < 10)			Strong evidence of genetic influence (10 $\le$ ${\mathrm{BF}}_{{\mathrm{h}}^{2}}$ < 100)			Strong evidence of genetic influence (${\mathrm{BF}}_{{\mathrm{h}}^{2}}$ $\ge$ 100)		
	${\mathrm{BF}}_{{\mathrm{h}}^{2}}$	h^2^	n	${\mathrm{BF}}_{{\mathrm{h}}^{2}}$	h^2^	n	${\mathrm{BF}}_{{\mathrm{h}}^{2}}$	h^2^	n
All	5.31 (1.62)	0.21 (0.03)	108	24.83 (18.09)	0.26 (0.05)	33	159.38 (15.27)	0.19 (0.04)	2
> 10 to $\le$ 25	5.03 (1.32)	0.20 (0.03)	6	26.92 (12.50)	0.23 (0.03)	4	170.18 (–)	0.16 (–)	1
> 25 to $\le$ 50	5.49 (1.79)	0.21 (0.03)	23	36.19 (31.80)	0.25 (0.04)	6	–	–	0
> 50 to $\le$ 75	5.46 (1.70)	0.21 (0.04)	44	21.23 (12.57)	0.28 (0.05)	12	148.58 (–)	0.22 (–)	1
> 75 to $\le$ 100	5.07 (1.48)	0.19 (0.03)	35	21.82 (14.73)	0.27 (0.07)	11	–	–	0

${\mathrm{BF}}_{{\mathrm{h}}^{2}}$ = Bayes factor of the model with additive genetic effects against the same model without additive genetic effects

**Table 4:** Mean (standard deviation) of Bayes factors and heritability estimates for OTU under genetic control adjusted with the ZIP model

Frequency (%)	Substantial evidence of genetic influence (3.2 $\le$ ${\mathrm{BF}}_{{\mathrm{h}}^{2}}$ < 10)			Strong evidence of genetic influence (10 $\le$ ${\mathrm{BF}}_{{\mathrm{h}}^{2}}$ < 100)		
	${\mathrm{BF}}_{{\mathrm{h}}^{2}}$	h^2^	n	${\mathrm{BF}}_{{\mathrm{h}}^{2}}$	h^2^	n
All	4.49 (1.73)	0.24 (0.04)	10	11.37 (–)	0.18 (–)	1
$\ge$ 5 to $\le$ 10	3.62 (0.35)	0.24 (0.01)	5	11.37 (–)	0.18 (–)	1
> 10 to $\le$ 25	5.37 (2.16)	0.23 (0.06)	5	–	–	0

${\mathrm{BF}}_{{\mathrm{h}}^{2}}$= Bayes factor of the model with additive genetic effects against the same model without additive genetic effects

The BF provided some level of evidence in favor of host genetics control for 154 of the 951 OTU analyzed. The BF between models with and without an additive genetic effect provided evidence of a substantial (${\mathrm{BF}}_{{\mathrm{h}}^{2}}$ $\ge$ 3.2) genetic control for 108 and 10 of the OTU that were better adjusted with the normal LMM and ZIP models, respectively. Evidence of a strong (10 $\le$ ${\mathrm{BF}}_{{\mathrm{h}}^{2}}$ < 100) genetic control was found for 33 and one OTU that were better adjusted with the normal LMM and ZIP models, respectively. Finally, decisive (${\mathrm{BF}}_{{\mathrm{h}}^{2 }}\ge$ 100) evidence of genetic control was found for two OTU that were better adjusted with the normal LMM. The taxonomic assignment of these two OTU revealed that one belongs to the genus *Bacteroides* and the other to the genus *Parabacteroides*, and the marginal posterior means (standard deviations) of their heritability estimates were 0.16 (0.07) and 0.22 (0.08), respectively (see Additional file 8: Table S8). Overall, the estimates of the heritability for these OTU were moderate (from 0.12 to 0.40). However, it should be noted that these estimates have large standard deviations as a consequence of our limited sample size. Nevertheless, it is worth mentioning that for 51 of the 154 OTU identified as being under genetic control, the probability that their heritability estimate is higher than 0.10 was equal or greater than 0.80.

Table 5 shows the marginal posterior means and standard deviations of the heritability estimates, together with the probability of these estimates being greater than 0.10, for relative abundances of genera and alpha-diversity indices. The BF provided evidence in favor of genetic control for the relative abundances of 10 genera. This evidence was substantial (${\mathrm{BF}}_{{\mathrm{h}}^{2}}$ $\ge$ 3.2) for the genera *Dehalobacterium*, *Epulopiscium*, *Methanobrevibacter*, *Butyricimonas*, *Odoribacter*, *Blautia* and *Oxalobacter*; and strong (10 $\le$ ${\mathrm{BF}}_{{\mathrm{h}}^{2}}$ < 100) for the genera *Phascolarctobacteirum*, *Bacteroides* and *Parabacteroides*. The estimates (marginal posterior means) of the heritability for these genera ranged from 0.17 to 0.35. The highest heritability estimates, accompanied by high BF values, were found for the genera *Bacteroides*, *Parabacteroides* and *Dehalobacterium*, for which the probability that their heritability estimates are higher than 0.10 was greater than 0.80. Although the evidence in favor of genetic control was strong for the genus *Phascolarctobacterium*, its heritability (0.19) estimate was not one of the highest (P(h^2^ > 0.1) = 0.73). However, 1/${\mathrm{BF}}_{{\mathrm{h}}^{2}}$ values higher than 3.2 were obtained only for 10 OTU and the genus *Coprococcus*, which are clearly not heritable.

**Table 5:** Bayes factors, marginal posterior means (standard deviations) of the heritability estimates for genera and alpha-diversity indices influenced by genetic effects

Trait	Mean (SD) h^2^	P (h^2^ > 0.1)	${\mathrm{BF}}_{{\mathrm{h}}^{2}}$
Genus *Methanobrevibacter*	0.21 (0.13)	0.79	7.75
Genus *Butyricimonas*	0.27 (0.19)	0.79	4.39
Genus *Odoribacter*	0.19 (0.13)	0.71	3.50
Genus *Bacteroides*	0.29 (0.17)	0.87	13.88
Genus *Parabacteroides*	0.35 (0.17)	0.91	31.15
Genus *Dehalobacterium*	0.29 (0.19)	0.83	8.62
Genus *Blautia*	0.20 (0.12)	0.78	7.01
Genus *Epulopiscium*	0.17 (0.11)	0.70	5.85
Genus *Phascolarctobacterium*	0.19 (0.12)	0.73	10.22
Genus *Oxalobacter*	0.21 (0.13)	0.78	6.12
Number of OTU observed	0.28 (0.17)	0.84	7.30
Shannon	0.18 (0.13)	0.70	3.41

SD: standard deviation; ${\mathrm{BF}}_{{\mathrm{h}}^{2}}$: Bayes factor of the model with additive genetic effects against the same model without additive genetic effects

Finally, regarding the traits that globally integrate the rabbit cecal microbiota, substantial evidence of genetic control was found for number of OTU observed and Shannon indices. The highest heritability estimate was found for the number of OTU observed index (h^2^ = 0.28; ${\mathrm{BF}}_{{\mathrm{h}}^{2}}$ = 7.30). The additive genetic background for these two traits was clearly demonstrated by a probability greater than 0.70 that their heritability estimates are higher than 0.10.

### Microbial traits influenced by the nursing environment

The marginal posterior means of litter variance ratio for OTU, categorized by frequency, which were better adjusted with the normal LMM according to DIC and for which the BF provided evidence in favor of a litter effect are summarized in Table 6, and the corresponding ones which were better adjusted with the ZIP model according to DIC are in Table 7.

**Table 6:** Mean (standard deviation) of Bayes factors and litter variance ratio estimates for OTU influenced by a litter effect adjusted with the normal LMM

Frequency (%)	Substantial evidence of litter influence (3.2 $\le$ ${\mathrm{BF}}_{{\mathrm{l}}^{2}}$ < 10)			Strong evidence of litter influence (10 $\le$ ${\mathrm{BF}}_{{\mathrm{l}}^{2}}$ < 100)			Decisive evidence of litter influence (${\mathrm{BF}}_{{\mathrm{l}}^{2}}$ $\ge$ 100)		
	${\mathrm{BF}}_{{\mathrm{l}}^{2}}$	l^2^	n	${\mathrm{BF}}_{{\mathrm{l}}^{2}}$	l^2^	n	${\mathrm{BF}}_{{\mathrm{l}}^{2}}$	l^2^	n
All	5.29 (1.79)	0.12 (0.01)	81	29.46 (22.86)	0.17 (0.02)	63	∞ (∞)	0.37 (0.15)	65
> 10 to $\le$ 25	4.08 (0.80)	0.12 (0.00)	2	10.09 (–)	0.14 (–)	1	∞ (∞)	0.54 (0.17)	9
> 25 to $\le$ 50	5.45(1.73)	0.12 (0.01)	16	25.23 (16.74)	0.17 (0.02)	13	∞ (∞)	0.40 (0.14)	19
> 50 to $\le$ 75	5.49 (1.90)	0.12 (0.01)	28	34.90 (26.65)	0.17 (0.02)	23	∞ (∞)	0.33 (0.12)	27
> 75 to $\le$ 100	5.12 (1.77)	0.12 (0.01)	35	27.51 (21.94)	0.17 (0.02)	26	7.98E4 (2.33E5)	0.27 (0.06)	10

${\mathrm{BF}}_{{\mathrm{l}}^{2}}$= Bayes factor of the model with litter effects against the same model without litter effects

**Table 7:** Mean (standard deviation) of Bayes factors and litter variance ratio estimates for OTU influenced by a litter effect adjusted with the ZIP model

Frequency (%)	Substantial evidence of litter influence (3.2 $\le$ ${\mathrm{BF}}_{{\mathrm{l}}^{2}}$ < 10)			Strong evidence of litter influence (10 $\le$ ${\mathrm{BF}}_{{\mathrm{l}}^{2}}$ < 100)		
	${\mathrm{BF}}_{{\mathrm{l}}^{2}}$	l^2^	N	${\mathrm{BF}}_{{\mathrm{l}}^{2}}$	l^2^	n
All	3.77 (0.46)	0.19 (0.00)	3	36.69 (23.57)	0.46 (0.14)	3
$\ge$ 5 to $\le$ 10	3.80 (0.64)	0.19 (0.00)	2	59.05 (–)	0.62 (–)	1
> 10 to $\le$ 25	3.70 (–)	0.19 (–)	1	25.51 (19.01)	0.38 (0.04)	2

${\mathrm{BF}}_{{\mathrm{l}}^{2}}$= Bayes factor of the model with litter effects against the same model without litter effects

The BF provided some level of evidence in favor of a litter effect for 215 of the 951 OTU analyzed. Six of them were better adjusted (lower DIC values) with the ZIP model and the remaining 209 were better adjusted with the LMM. BF values between models with and without a litter effect provided evidence of a substantial (${\mathrm{BF}}_{{\mathrm{l}}^{2}}$ $\ge$ 3.2) litter effect for 81 and three OTU that were better adjusted with the normal LMM and the ZIP model, respectively. Strong evidence (10 $\le$ ${\mathrm{BF}}_{{\mathrm{l}}^{2}}$ < 100) of a litter effect was found for 63 and three OTU that were better adjusted with the normal LMM and the ZIP model, respectively. Finally, decisive (${\mathrm{BF}}_{{\mathrm{l}}^{2}}\ge$ 100) evidence of a litter effect was found for 65 OTU that were better adjusted with the normal LMM. The taxonomic assignment of these OTU revealed that most of them belong to the genera *Parabacteroides*, *Phascolarctobacterium*, and the species *eggerthii* and *fragilis* of the genus *Bacteroides*. Overall, the marginal posterior means of the litter variance ratio ranged from 0.12 to 0.19 (Tables 6 and 7) but the estimates for this ratio reached values ranging from 0.37 to 0.54 for the OTU for which large BF values were observed (see Additional file 9: Table S9). Eighty-nine of the 215 OTU that were declared to be influenced by litter effects had a probability equal or greater than 0.80 that their litter variance ratio be greater than 0.10. The marginal posterior means of the litter variance ratio were greater than 0.50 for 12 OTU of which six belong to the genus *Bacteroides*, four to the genus *Phascolarctobacterium*, one to the genus *Parabacteroides* and one to the genus *Rikenella* (see Additional file 9: Table S9). It should be mentioned that the 1/${\mathrm{BF}}_{{\mathrm{l}}^{2}}$ values were greater than 3.2 for 107 OTU, which are not influenced by a litter effect.

The marginal posterior means of the litter variance ratio, together with the associated probability of these estimates being greater than 0.10, for relative abundances of genera and alpha-diversity indices are in Table 8. The number of OTU observed index was found to be influenced by the nursing environment (l^2^ = 0.17; ${\mathrm{BF}}_{{\mathrm{l}}^{2}}$ = 15.55). The BF also provided evidence of a litter effect for 10 genera. Very strong evidence of a litter effect was found for the genera *Butyricimonas* (l^2^ = 0.28), *Bacteroides* (l^2^ = 0.27), *Parabacteroides* (l^2^ = 0.47), *Rikenella* (l^2^ = 0.32), *Dehalobacterium* (l^2^ = 0.37) and *Phascolarctobacterium* (l^2^ = 0.66) with decisive BF values (${\mathrm{BF}}_{{\mathrm{l}}^{2}} \ge$ 100) and P(l^2^ > 0.1) = 0.96. However, the genera *Coprococcus*, *rc4-4* and *Faecalibacterium* are not influenced by a litter effect (1/${\mathrm{BF}}_{{\mathrm{l}}^{2}}$ > 3.2).

**Table 8:** Bayes factors, marginal posterior means (standard deviations) of litter variance ratio for genera and alpha-diversity indices influenced by litter effects

Trait	Mean (SD) l^2^	P(l^2^ > 0.1)	${\mathrm{BF}}_{{\mathrm{l}}^{2}}$
Genus *Butyricimonas*	0.28 (0.10)	0.96	728.23
Genus *Odoribacter*	0.14 (0.08)	0.64	7.21
Genus *Bacteroides*	0.27 (0.09)	0.97	809.53
Genus *Parabacteroides*	0.47 (0.10)	1.00	1.50E11
Genus *Rikenella*	0.32 (0.08)	1.00	3.67E4
Genus *Dehalobacterium*	0.37 (0.10)	1.00	9.66E4
Genus *Anaerofustis*	0.15 (0.08)	0.68	8.80
Genus *Epulopiscium*	0.12 (0.07)	0.58	4.52
Genus *Phascolarctobacterium*	0.66 (0.07)	1.00	∞
Genus *Desulfovibrio*	0.17 (0.09)	0.78	16.53
Genus *Campylobacter*	0.16 (0.08)	0.73	11.91
Number of OTU observed	0.17 (0.09)	0.75	15.55

SD: standard deviation; ${\mathrm{BF}}_{{\mathrm{l}}^{2}}$: Bayes factor of the model with litter effects against the same model without litter effects

## Discussion

The influence of many external factors on the rabbit cecal microbial composition and diversity is well documented [4–8], but not that of the host genetics. To shed light on this, we report the heritability estimates, together with the litter and cage (results for cage effects can be found in Additional file 7: Tables S5, S6, and S7) variance ratios estimates, for microbial traits that were fitted with LMM and ZIP mixed models. Moreover, we have assessed the statistical relevance of these estimates through BF.

Previous studies in humans and different livestock species have pointed out the existence of host genetics control of the gut microbiota, but, to our knowledge, this has not been investigated in the rabbit. For the first time, we have evaluated the host genetics, litter, and cage effects on the microbial composition of the rabbit cecum, which is the organ that contains the greatest microbial diversity and complexity [2]. In this study, we defined a set of 984 microbial traits that represent cecal microbial composition and diversity with different levels of complexity. Although genus assignment of every queried sequence would be the desired outcome, it should be noted that the resolution of the 16S rDNA locus with the MiSeq technology used in this study allowed annotation of only 21% of all the sequences at the genus level. Nevertheless, given the importance of the functional roles played by bacteria that can be assigned at the genus level, it is relevant to provide estimates that inform about the influence of genetic, litter and cage effects for groups of reads that belong to the same genus.

The CSS-normalized abundances of 951 OTU can be considered as the most specific level of defining a microbial community, but their frequencies vary widely among samples. This means that some OTU are present in all or almost all the animals (i.e., core OTU), while others are only detected in some animals. The distribution of the OTU that are present only in a small percentage of the analyzed animals was clearly far from a normal distribution and, thus not surprisingly, these were better adjusted with the ZIP model. In spite of this, all the microbial traits analyzed in this study with a frequency higher than 25% were better adjusted with the normal LMM model according to DIC. For the traits with a clear excess of zero counts (i.e., having a frequency among samples lower than 15%), DIC clearly favored the ZIP model. Previous microbiome studies have also modeled microbiome data with the ZIP model to account for the excess of zero counts of many taxa that are rare and only detected in a small proportion of the samples [24, 41]. Such studies argue that the application of a conventional linear model is inappropriate for zero-inflated data. In our study, the ZIP model performed better than the LMM only for the microbial traits with a very marked excess of zero counts. However, it should be noted that our choice of the ZIP model, as an alternative to the LMM, was based only on its ability to handle the excess of zero counts, and not on whether the non-zero inflated component of the model was properly adjusted to a Poisson distribution. A lack of adjustment of transformed CSS records to the Poisson model could perfectly explain the fact that the ZIP model performed better than the LMM only for the microbial traits that have a very large excess of zero counts. Although we have derived some biological conclusions from the application of our models, we are aware of the limitation due to the application of LMM on records with a marked excess of zero counts. Handling such issues of CSS counts remains a challenge.

The BF provided evidence in favor of host genetics control for 34% and 16% of the genera and OTU present in the rabbit cecum, respectively. These results are in line with the analysis of heritability estimates conducted in humans by Goodrich et al. [42] who found evidence of a genetic control for 10% of their 945 identified taxa, and also with an assessment in cattle of the host genetics influence on the rumen microbiota [13] that showed that 34% of the microbial taxa analyzed (from genus to phylum levels) were heritable. Our heritability estimates for the relative abundances of the genera and OTU that were declared to be under host genetics control by the BF were moderate. This is also in agreement with earlier studies in humans and other livestock species. However, it is noteworthy that these studies suggested that the main heritable bacteria belong to the phylum *Firmicutes*, whereas taxa encompassed by the phylum *Bacteroidetes* are generally not heritable [13, 43]. A discussion of the results regarding the effects of host genetics, litter, and cage on taxa that are encompassed by the phyla *Bacteroidetes* and *Firmicutes*, as well as on microbial alpha-diversity indices, will be presented below.

In our study, according to the BF, the strongest evidence of host genetics control concerned two OTU that are taxonomically assigned to the genera *Bacteroides* and *Parabacteroides*, which are both part of the phylum *Bacteroidetes*. Moreover, the heritability estimates were highest for these two genera: 0.35 and 0.29 for *Parabacteroides* and *Bacteroides*, respectively. In pigs, Chen et al. [17] and Bergamaschi et al. [44] also reported some heritable taxa that belong to the phylum *Bacteroidetes*. The species that belong to the genera *Bacteroides* and *Parabacteroides* are anaerobic Gram-negative bacteria, which are involved in the degradation of plant polysaccharides and amino acid fermentation, amino acid transport, and cell motility in the gastrointestinal microbiota of the growing rabbit [45, 46]. Although the BF and our heritability estimates for the genera *Bacteroides* and *Parabacteroides* clearly reveal the existence of a host genetics control, the environmental effect of litter has a profound impact on the relative abundances of both genera (l^2^ *Parabacteroides* = 0.47; l^2^ *Bacteroides* = 0.27). The nursing environment provided by the mother and the siblings also has an important impact on the relative abundance of the genus *Rikenella* (l^2^ *Rikenella* = 0.32), which is also part of the phylum *Bacteroidetes*. Litter effects play an important role on phenotypic traits related to rabbit growth and feed efficiency [47]. The microbial colonization of the gastrointestinal tract of mammals is considered to occur immediately after birth when the newborns acquire their immature microbiota from a combination of maternal and external microbes [3, 48]. The impact of the nursing environment on the relative abundances of these genera still prevails at slaughter age when we collected the cecal samples for this study. Remarkably, the ratio of phenotypic variance due to litter effects exceeds 0.50 for six OTU that belong to the genus *Bacteroides* and for one OTU that is taxonomically assigned to the genus *Parabacteroides*. It is also worth noting that cage seems to have an important effect in the relative abundances of some members of the family *S24-7*. Bacteria within this family, which is part of the order *Bacteroidales*, have been shown to be dominant in the mouse gut microbiota and have been detected in the gastrointestinal tract of different mammals. The classification of this family remains ambiguous because its members have not been cultured, but the functional analysis conducted by Lagkouvardos et al. [49] renamed it as the family *Muribaculaceae*. A recent study on mice showed that members of the family *Muribaculaceae* are major mucin monosaccharide foragers in the gut [50].

High heritability estimates, accompanied by strong evidence of host genetics control based on the BF, were also estimated for the genera *Dehalobacterium* (h^2^ = 0.29) and *Butyricimonas* (h^2^ = 0.27). Both genera belong to the phylum *Firmicutes* and have been previously reported as heritable in humans [42, 51]. Such studies reported a module of co-occurring heritable families within which the family *Christensenellaceae* was the hub (i.e., the node connected to most other nodes) connected to heritable families *Methanobacteriaceae* and *Dehalobacteriaceae*. Interestingly, we also found substantial evidence of genetic control for the genus *Methanobrevibacter*, which is encompassed by the family *Methanobacteriaceae*. *Methanobrevibacter* is the single genus of the phylum *Euryarchaeota* that was detected in the rabbit cecum. It encompasses different hydrogenotrophic methane-producing species whose abundances have been associated with single-nucleotide polymorphisms that are located in a long noncoding RNA, however, this link remains uncertain [52]. In addition, taxa belonging to the family *Methanobacteriaceae* were reported to have heritability estimates higher than 0.50 in a beef cattle population [53]. It is worth noting that we also found heritability estimates statistically higher than zero for the genera *Blautia* and *Odoribacter*, which is consistent with previous results in humans [54, 55].

Our results revealed an important litter effect on the relative abundances of the genera *Butyricimonas* (l^2^ = 0.28), *Dehalobacterium* (l^2^ = 0.37) and *Phascolarctobacterium* (l^2^ = 0.66). Estimates of the heritability and BF also suggested a genetic control for these three genera, but the effect exerted by the nursing environment seems to be stronger. Regarding the role played by cage environmental effects, they were strong for some species encompassed by the genus *Ruminococcus*. On the contrary, host genetics and litter effects do not seem to have any relevant influence on this genus. However, La Reau et al. [56] and Li et al. [13] found that the abundance of the genus *Ruminococcus* was influenced by the host genetics. Remarkably, this genus displays a large diversity and, our results identified four OTU that are taxonomically assigned as *Ruminococcus* and are clearly influenced by the host genetics.

On a global scale, our results suggest that a substantial part of the cecal microbial variability is under host genetics control since the BF provided evidence in favor of genetic control for the number of OTU observed and Shannon indices. In line with previous heritability assessments of alpha-diversity in pigs [18, 44] and humans [43], we have found a clear genetic background for the number of OTU observed index (h^2^ = 0.28). Microbial complexity can be summarized into alpha-diversity indices, which are heritable traits that could potentially be incorporated into breeding programs if an association with a production trait of economic interest was demonstrated. Nonetheless, it is important to bear in mind that alpha-diversity at weaning might not be an accurate predictor of diversity at later stages in rabbit life. Rabbit cecum hosts a rich and complex microbial ecosystem that is shaped by many non-genetic factors, however, a significant proportion of the microbial traits analyzed in this study showed moderate heritabilities. Although the cecal samples analyzed here were collected from nearly adult rabbits, these estimates should be interpreted with caution since microbial composition changes as the animal grows and does not stabilize until the animal reaches adulthood. As we have stated, recent studies in different livestock species have attempted to dissect the host genetics control of gut microbiota but without paying enough attention to non-genetic factors, such as litter or cage effects, which can be even more relevant than the additive genetic effects.

For the first time, we have evaluated the role played by host genetics, litter and cage effects on a set of traits that attempt to represent the rabbit cecal microbiota at different levels of complexity. Understanding how these effects influence the intestinal microbiota is relevant from a biological perspective. One example is the genus *Methanobrevibacter*, which is clearly heritable and seems to be linked to methane emissions. The host genetics control for methane emissions and the relative abundance of this genus would offer the possibility to alter the microbial composition through selection and to breed for rabbits with a reduced impact on climate. Although selection to reduce this genus could be recommended, it would only account for a certain part of the methane emissions. Moreover, members of this genus could be beneficial for other relevant traits, thus selecting for a given trait via the microbiota might result in negative responses for other traits of interest. A direct selection somehow guarantees a balanced modification of all the elements involved in the metabolic pathway of the trait.

Finally, it must be noted that the mechanisms underlying the host genetics control on cecal microbiota remain unknown. Future genome-wide association studies with large datasets are necessary to identify the host genomic regions that are involved in the control of the overall microbial diversity and abundances of specific taxa.

## Conclusions

The Bayesian analysis of a set of 984 microbial traits conducted in this study with LMM and ZIP mixed models has allowed us to dissect the additive genetic, litter and cage effects on different levels of complexity of the rabbit cecal microbiota based on BF. Fitting these microbial traits with a LMM model was preferable except for the analyses of the CSS-normalized abundances of rare OTU that are characterized by a marked excess of zero counts and were better adjusted with the ZIP model. The calculation of BF as an assessment tool of the statistical relevance of the estimates of heritability, litter and cage variance ratios has allowed us to unravel the different levels of evidence in favor of these effects on the global cecal microbial composition and on an important proportion of OTU and relative abundances of genera. We found a strong influence of the host genetics and the nursing environment for members of the genera *Bacteroides* and *Parabacteroides*, and of cage effects for the family *S24-7* and the genus *Ruminococcus*. Our findings provide support that the host genetics, cage and nursing environment contribute to the variation of the rabbit cecal microbial composition, but functional and genome-wide association studies are needed to decipher the underlying mechanisms.

## Supplementary Information

**Additional file 1: Table S1.** Metadata associated with the 425 rabbit cecal samples analyzed in this study.

**Additional file 2: Table S2.** Table of filtered and CSS-normalized OTU.

**Additional file 3: Table S3.** Taxonomic assignment of representative sequences of each OTU included in Additional file 2: Table S2.

**Additional file 4: Figure S1.** Geweke z-statistics for genetic, litter and cage variance ratios computed for the microbial traits that were better adjusted with the normal linear mixed model.

**Additional file 5: Figure S2.** Geweke z-statistics for genetic, litter and cage variance ratios computed for the microbial traits that were better adjusted with the zero-inflated Poisson model.

**Additional file 6: Table S4.** Deviance information criterion (DIC) values computed for each microbial trait with the normal linear mixed model and the zero-inflated Poisson model.

**Additional file 7: Table S5.** Mean (standard deviation) of Bayes factors and cage variance ratio estimates for OTU that are influenced by cage effects and adjusted with the normal LMM. **Table S6.** Mean (standard deviation) of Bayes factor and cage variance ratio estimates for OTU that are influenced by cage effect and adjusted with the ZIP model. **Tables S7.** Bayes factors, marginal posterior means (standard deviations) of cage variance ratio for genera and alpha-diversity indices that are influenced by cage effects.

**Additional file 8: Table S8.** Bayes factors, marginal posterior means and standard deviations of heritability estimates for OTU under genetic control together with the associated probability of these estimates being higher than 0.10. The frequency among the rabbit samples and the taxonomic assignment are also provided for each OTU.

**Additional file 9: Table S9.** Bayes factors, marginal posterior means and standard deviations of litter variance ratio for OTU influenced by litter effects together with the associated probability of these estimates being higher than 0.10. The frequency among the rabbit samples and the taxonomic assignment are also provided for each OTU.

**Additional file 10: Table S10.** Bayes factors, marginal posterior means and standard deviations of cage variance ratio for OTU influenced by cage effects together with the associated probability of these estimates being higher than 0.10. The frequency among the rabbit samples and the taxonomic assignment are also provided for each OTU.

## Data Availability

The raw sequence data were deposited in the sequence read archive of NCBI under the accession number SRP186982 (BioProject PRJNA524130). Metadata, the filtered and CSS-normalized OTU table and corresponding taxonomic assignments are all included in Additional file 1: Table S1, Additional file 2: Table S2 and Additional file 3: Table S3, respectively. These data are available in Qiita (https://qiita.ucsd.edu/) [57] under study ID 14485.
