# Supplementary figures and images for: Use of Bayes factors to evaluate the effects of host genetics, litter and cage on the rabbit cecal microbiota
Source: Genet Sel Evol. 2022 Jun 27;54:46. doi: 10.1186/s12711-022-00738-2 (PMC9235133; doi:10.1186/s12711-022-00738-2)

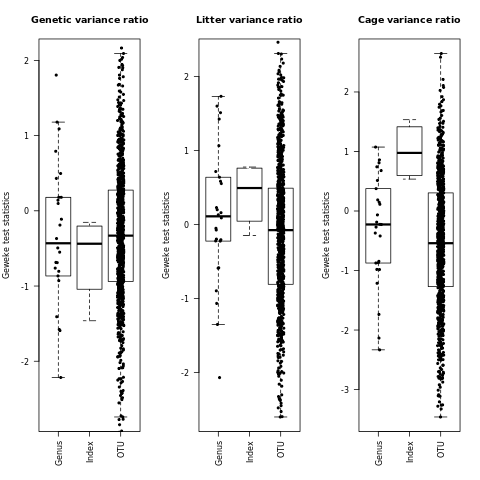

Supplement: Supplementary file 4 — Additional file 4: Figure S1. Geweke z-statistics for genetic, litter and cage variance ratios computed for the microbial traits that were better adjusted with the normal linear mixed model. [file 12711_2022_738_MOESM4_ESM.tiff]

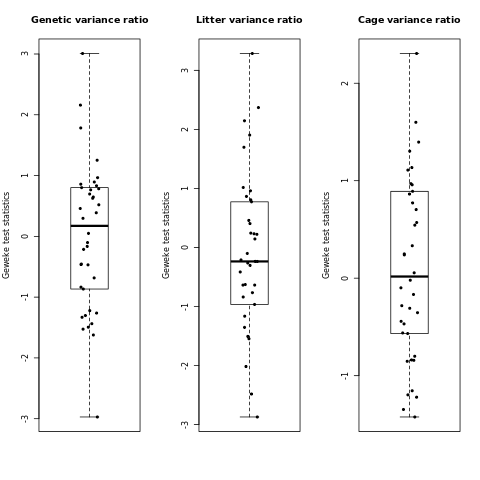

Supplement: Supplementary file 5 — Additional file 5: Figure S2. Geweke z-statistics for genetic, litter and cage variance ratios computed for the microbial traits that were better adjusted with the zero-inflated Poisson model. [file 12711_2022_738_MOESM5_ESM.tiff]
