# Supplementary material for: Use of Bayes factors to evaluate the effects of host genetics, litter and cage on the rabbit cecal microbiota
Source: Genet Sel Evol. 2022 Jun 27;54:46. doi: 10.1186/s12711-022-00738-2 (PMC9235133; doi:10.1186/s12711-022-00738-2)
Supplement: Supplementary file 7 — Additional file 7: Table S5. Mean (standard deviation) of Bayes factors and cage variance ratio estimates for OTU that are influenced by cage effects and adjusted with the normal LMM. Table S6. Mean (standard deviation) of Bayes factor and cage variance ratio estimates for OTU that are influenced by cage effect and adjusted with the ZIP model. Tables S7. Bayes factors, marginal posterior means (standard deviations) of cage variance ratio for genera and alpha-diversity indices that are influenced by cage effects. [file 12711_2022_738_MOESM7_ESM.docx]

**Microbial traits influenced by the cage**

Additional file 10 Table S10 includes Bayes factors, marginal posterior means and standard deviations of the cage variance ratio for OTU influenced by cage effects together with the associated probability of these estimates being higher than 0.10. The taxonomic assignment of the representative sequences of such OTU and their frequency among the rabbit samples are also shown in this file. Additional file 7 Table S5 shows the marginal posterior means of cage variance ratio for OTU, categorized by frequency, which were better adjusted with the normal LMM and for which the BF provided evidence in favor of a cage influence. Similarly, Additional file 7 Table S6 includes the same information for OTU which were better adjusted with the ZIP model and for which the BF provided evidence in favor of a cage influence.

The BF provided some level of evidence in favor of a cage effect for 143 OTU which were better adjusted with the normal LMM of which 79, 47 and 17 showed substantial, strong and decisive, respectively, evidence. While the BF provided substantial and strong evidence in favor of a cage effect for four and one OTU, respectively, which were better adjusted with the ZIP model. The taxonomic assignment of these OTU revealed that many of them belong to the families *S24-7* and *Ruminococcaceae* [see Additional file 10 Table S10]. Overall, the marginal posterior means of the cage variance ratio ranged from 0.11 to 0.24 (Additional file 7 Tables S5 and S6) but three OTU for which large BF values were calculated reached cage variance ratio estimates up to 0.46 [see Additional file 10 Table S10]. Two of these OTU were assigned to the family *S24-7* [see Additional file 10 Table S10]. It should be noted that 1/$\mathrm{BF}_{c^{2}}$ values higher than 3.2 were obtained for 130 OTU, which are not influenced by a cage effect.

**Additional file 7 Table S5 Mean (standard deviation) of Bayes factor and cage variance ratio estimates for OTU influenced by a cage effect adjusted with the normal LMM.**

| **Frequency (%)** | **Substantial evidence of cage influence (3.2** $\leq$ $\mathbf{BF}_{\mathbf{c}^{\mathbf{2}}}$ **<10)** | | | **Strong evidence of cage influence (10** $\leq$ $\mathbf{BF}_{\mathbf{c}^{\mathbf{2}}}$ **< 100)** | | | **Decisive evidence of cage influence (**$\mathbf{BF}_{\mathbf{c}^{\mathbf{2}}}$ $\boldsymbol{\geq}$ **100)** | | |
| --- | --- | --- | --- | --- | --- | --- | --- | --- | --- |
|  | $\mathbf{BF}_{\mathbf{c}^{\mathbf{2}}}$ | **c^2^** | **n** | $\mathbf{BF}_{\mathbf{c}^{\mathbf{2}}}$ | **c^2^** | **n** | $\mathbf{BF}_{\mathbf{c}^{\mathbf{2}}}$ | **c^2^** | **n** |
| All | 5.68 (1.94) | 0.11 (0.01) | 79 | 27.92 (22.14) | 0.14 (0.02) | 47 | 6.93E7 (2.81E8) | 0.21 (0.07) | 17 |
| > 10 to $\leq$ 25 | 3.34 (-) | 0.11 (-) | 1 | 14.79 (-) | 0.14 (-) | 1 | 497.12 (-) | 0.08 (-) | 1 |
| > 25 to $\leq$ 50 | 5.82 (1.33) | 0.10 (0.02) | 14 | 36.98 (22.55) | 0.14 (0.02) | 7 | 2.32E8 (5.19E8) | 0.24 (0.09) | 5 |
| > 50 to $\leq$ 75 | 5.62 (2.13) | 0.11 (0.01) | 21 | 23.82 (21.38) | 0.14 (0.02) | 17 | 2.71E6 (6.63E6) | 0.22 (0.05) | 6 |
| > 75 to $\leq$ 100 | 5.72 (2.04) | 0.11 (0.01) | 43 | 28.80 (23.04) | 0.15 (0.01) | 22 | 700.53 (684.16) | 0.20 (0.02) | 5 |

$\mathrm{BF}_{c^{2}}$: Bayes factor of the model with cage effects against the same model without cage effects

**Additional file 7 Table S6 Mean (standard deviation) of Bayes factor and cage variance ratio estimates for OTU influenced by a cage effect adjusted with the ZIP model**

| **Frequency (%)** | **Substantial evidence of cage influence (3.2** $\boldsymbol{\leq}$ $\mathbf{BF}_{\mathbf{c}^{\mathbf{2}}}$ **< 10)** | | | **Strong evidence of cage influence (10** $\boldsymbol{\leq}$ $\mathbf{BF}_{\mathbf{c}^{\mathbf{2}}}$ **< 100)** | |  |
| --- | --- | --- | --- | --- | --- | --- |
|  | $\mathbf{BF}_{\mathbf{c}^{\mathbf{2}}}$ | **c^2^** | **n** | $\mathbf{BF}_{\mathbf{c}^{\mathbf{2}}}$ | **c^2^** | **n** |
| All | 3.93 (0.70) | 0.16 (0.05) | 4 | 37.39 (-) | 0.46 (-) | 1 |
| $\geq$5 to $\leq$10 | 4.53 (0.04) | 0.14 (0.08) | 2 | - | - | 0 |
| >10 to $\leq$25 | 3.33 (0.16) | 0.17 (0.01) | 2 | 37.39 (-) | 0.46 (-) | 1 |

$\mathrm{BF}_{c^{2}}$: Bayes factor of the model with cage effects against the same model without cage effects.

Finally, the marginal posterior means of the cage variance ratio, together with the associated probability of this ratio being greater than 0.10, for relative abundances of genera and the traits defined to globally integrate the rabbit cecal microbiota are in Additional file 7 Table S7. The BF provided evidence of a cage effect for three genera whose marginal posterior means of the cage variance ratio ranged from 0.11 to 0.22. Although these estimates are accompanied by large standard deviations as a consequence of our limited sample size, a patent cage influence was demonstrated for the genus *Ruminococcus* (c^2^ = 0.22; BF = 648.80; P(c^2^ > 0.1) = 0.95).

**Additional file 7 Table S7 Bayes factors, marginal posterior means (standard deviations) of cage variance ratio for genera and alpha-diversity indices influenced by cage effects.**

| **Trait** | **Mean (SD) c^2^** | **P(c^2^ > 0.1)** | $\mathbf{BF}_{\mathbf{c}^{\mathbf{2}}}$ |
| --- | --- | --- | --- |
| Genus *Ruminococcus* | 0.22 (0.07) | 0.95 | 648.80 |
| Genus *Dorea* | 0.11 (0.07) | 0.52 | 3.23 |
| Genus *Faecalibacterium* | 0.11 (0.06) | 0.50 | 3.31 |

SD: standard deviation; $\mathrm{BF}_{c^{2}}$: Bayes factor of the model with cage effects against the same model without cage effects.
